# Efficacy and safety of early YAG laser vitreolysis for symptomatic vitreous floaters: the study protocol for a randomized clinical trial

**DOI:** 10.1186/s13063-024-07924-1

**Published:** 2024-01-13

**Authors:** Zhou Hangshuai, Jin Yanhua, Zhou Yao, Zhao Guangjin, Wu Hongyan, Chen Fanlian

**Affiliations:** Department of Ophthalmology, Dongyang People’s Hospital, No. 60 Wu Ning West Road, Dongyang, Zhejiang China

**Keywords:** Vitreous floaters, Vitreolysis, Nd: YAG laser

## Abstract

**Background:**

Vitreous floaters are a common ocular condition that affects individuals of all ages. Although vitreous floaters are typically benign, they can significantly impair visual acuity and quality of life. Laser vitreolysis, which uses an Nd: YAG laser to vaporize collagenous vitreous opacities, is increasingly being used as a treatment option. However, there is currently a lack of evidence regarding its efficacy and the appropriate timing of its application. This study aims to evaluate the efficacy and safety of early intervention with YAG laser vitreolysis in treating symptomatic vitreous floaters.

**Methods:**

The present study is a randomized, controlled, double-blind clinical trial. A total of 70 participants with symptomatic floaters for 1 month were prospectively recruited. These participants will be randomly assigned to two groups, with 35 individuals in each group: the early treatment group and the delayed treatment group. Participants assigned to the early treatment group will undergo YAG laser vitreolysis immediately, followed by a sham laser treatment 3 months later. On the other hand, participants assigned to the delayed treatment group will receive a sham laser treatment and then undergo YAG laser vitreolysis 3 months later. The follow-up time points will be 1, 3, 6, and 12 months from randomization. Primary outcomes will be participants’ self-reported improvement in visual disturbance on a scale of 1 to 10 and their scores on the National Eye Institute Visual Functioning Questionnaire 25 (NEI VFQ-25). Secondary outcomes will be an objective evaluation of the effectiveness of the treatment in reducing vitreous floaters through OCT and fundus photography and tracking any adverse events related to the eyes or overall health.

**Discussion:**

This clinical trial aims to evaluate the effectiveness of YAG laser vitreolysis in treating symptomatic vitreous floaters and assess the safety of performing early intervention with YAG laser vitreolysis.

**Trial registration:**

ClinicalTrials.gov NCT05800353. Registered on 10 March 2023.

**Supplementary Information:**

The online version contains supplementary material available at 10.1186/s13063-024-07924-1.

## Background

Floaters are visual disturbances that occur when the vitreous in the eye becomes cloudy, which can happen when the posterior vitreous detaches. Vitreous floaters can cause serious visual discomfort symptoms [1, 2]. In an electronic survey that recruited 603 smartphone users, 76% of the participants reported seeing floaters, while 33% complained of noticeable visual impairment because of them [3]. In the past, observation and vitrectomy were the main methods for treating vitreous floaters. Although the majority of vitreous floaters patients are currently recommended for clinical observation, many still cannot tolerate the related visual symptoms and seek further treatment. Vitrectomy has a definite therapeutic effect, but as an invasive procedure, it has relatively more complications and higher costs and is still not accepted by most clinical practitioners [4]. In recent years, ND: YAG laser vitreolysis has gained popularity as a fast, relatively inexpensive, and less invasive method for treating vitreous opacities and is increasingly being considered a viable option for managing vitreous floaters [5].

In 2002, Delaney et al. [6] applied YAG laser to treat 38 patients with vitreous floaters, and the results showed that 38% of patients experienced a moderate or greater subjective improvement in symptoms. Among them, 11 patients were unsatisfied with the outcome of the YAG laser and subsequently underwent vitrectomy. In 2017, Shah et al. [7] conducted the first randomized controlled trial comparing YAG laser and sham laser treatment for vitreous floaters. The results showed that 53% of patients in the true laser group had a significant subjective improvement in symptoms, while there was no significant improvement in the sham laser group. There were no significant differences in adverse reactions between the two groups, further confirming the effectiveness and safety of the YAG laser in the treatment of vitreous floaters. Shah et al. believed that Delaney used a low energy intensity when treating vitreous floaters with the YAG laser, only cutting rather than vaporizing the opacities, which resulted in poor therapeutic effects. A recent long-term follow-up study showed that the therapeutic effect of the YAG laser could be maintained for up to 18 months [8].

Although numerous studies have demonstrated the efficacy and safety of YAG laser vitreolysis, there remains controversy among some researchers regarding its widespread clinical use [9]. They argue that, as with any new treatment modality, moderate skepticism is warranted until further research demonstrates a favorable risk/benefit ratio. Additionally, Shah’s research found that objective improvement in patients’ vitreous floaters was 94%, while subjective improvement was only 53%, indicating a clear disconnect between objective and subjective responses that must be taken into account. On the efficacy front, the majority of current research does not rule out the placebo effect, as symptoms of vitreous opacities themselves may improve with gravity and neural adaptation. Complications of YAG laser are also worth paying attention to, such as lens damage, retinal hemorrhage, retinal vein occlusion, and postoperative high intraocular pressure [10–13]. To reduce intraoperative complications, YAG laser vitreolysis is recommended for patients with stable Weiss rings, with floaters located more than 2 mm away from the retina and more than 5 mm away from the lens [14].

To reduce intraoperative complications, current randomized controlled trials require inclusion criteria of patients with vitreous floaters lasting more than 6 months and completed posterior vitreous detachment [7, 15], which is not clinically practical. Many patients require intervention in the early stages of symptoms. There is a lack of research on the safety and efficacy of early YAG laser vitreolysis. Therefore, we undertook this clinical study to uncover that early YAG laser vitreolysis is both a safe and effective treatment option for vitreous floaters.

## Methods/design

**Fig. 1:**
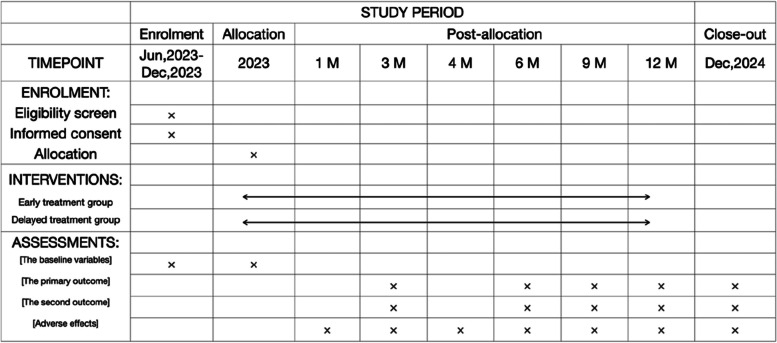
Standardized Protocol Items: Recommendations for Interventional Trials (SPIRIT)

**Fig. 2:**
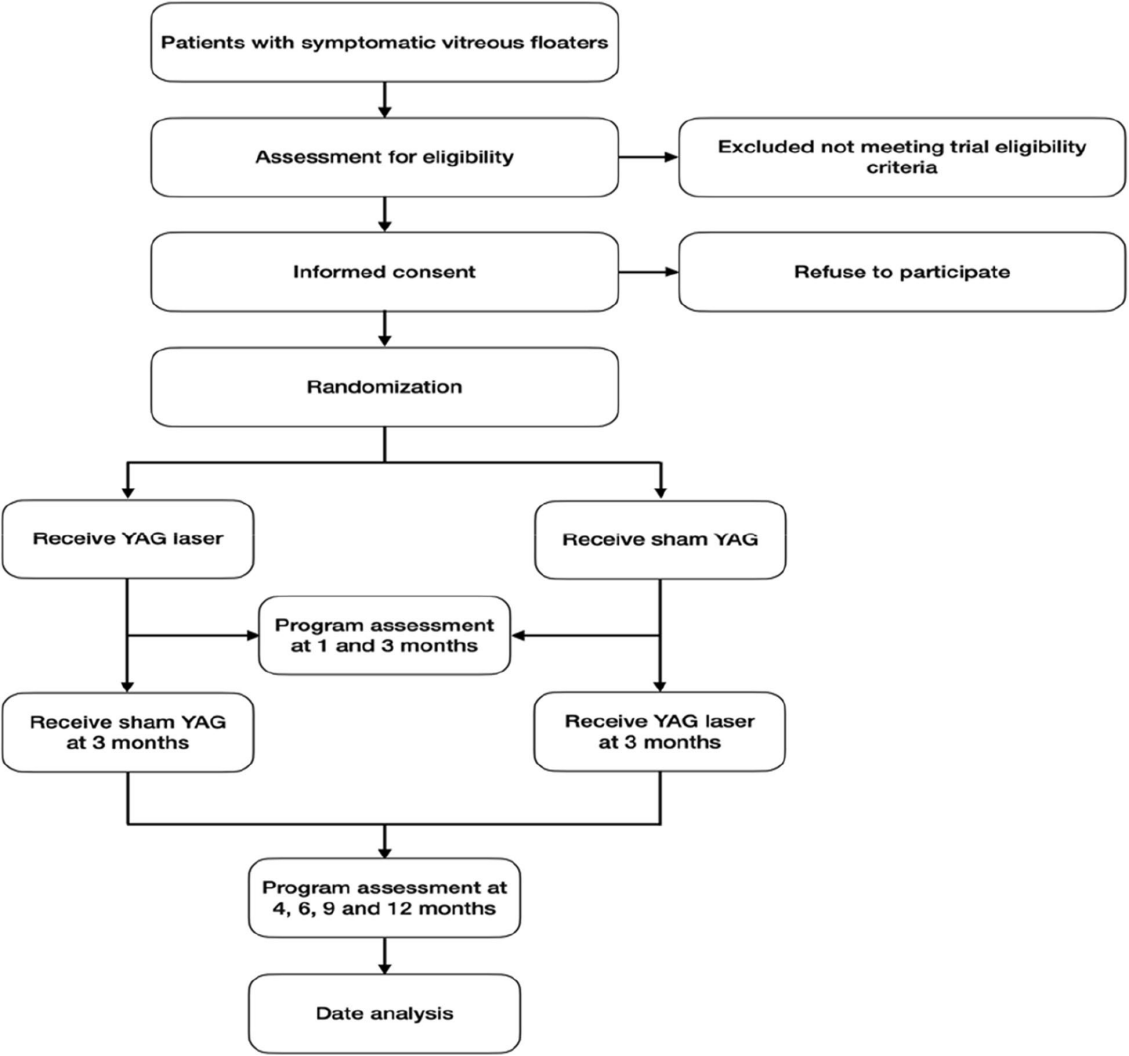
Flow of participants

The present prospective, randomized, controlled, double-blind, non-inferiority clinical trial aimed to evaluate the efficacy and safety of early YAG laser vitreolysis in treating symptomatic vitreous floaters. The clinical trial is presently being undertaken at Dongyang People’s Hospital, China. Ethical approval was obtained from the institutional ethics committee at the Dongyang People’s Hospital. Before enrollment, written informed consent was collected from the participants. The schedule for enrollment, intervention, data collection, and assessment was by the Standardized Protocol Items: Recommendations for Interventional Trials (SPIRIT) guidelines (see Fig. 1). The study flow schedule is depicted in Fig. 2. We followed the SPIRIT reporting guidelines during the drafting of this article [16].

### Participant eligibility

#### Inclusion criteria

Patients with a Weiss ring duration of less than 10 days and without additional eye complications will receive instructions to return to the hospital 1 month after the onset of symptoms. Subsequently, participants experiencing symptomatic floaters for precisely 1 month (28 to 32 days after the onset of symptoms) will be eligible for enrollment in the study. To be eligible for the study, the patient must rate their visual disturbance caused by floaters as at least 4 on a 0–10 scale, with 0 being no symptoms and 10 being debilitating symptoms. The symptomatic Weiss ring must be at least 2 mm from the retina and 5 mm from the posterior capsule of the crystalline lens, as measured on the B-scan. There is no minimum required distance from the intraocular lens for patients who have had cataract surgery (pseudophakic patients). The patient must be able to position themselves for the YAG laser procedure and accept the risks associated with the procedure, including but not limited to retinal detachment, intraocular hemorrhage, retinal damage, cataract formation, optic nerve damage, inflammation, and irreversible loss of vision. Additionally, the patient must be willing and able to comply with clinic visits and study-related procedures. If the patient has symptoms in both eyes, only one eye can be included in the study and randomized.

#### Exclusion criteria

The exclusion criteria are as follows: history of a retinal detachment, retinal tear, or uveitis in the study eye; history of macular edema, diabetic retinopathy, retinal vein occlusion, or aphakia in the study eye; history of glaucoma or high intraocular pressure, defined as having undergone glaucoma surgery in the eye being studied, or currently using two or more topical glaucoma medications in the eye being studied.

### Randomization and masking

In this study, a stratified randomization method will be used to allocate participants to two groups in a 1:1 ratio, using SPSS v26.0 (SPSS Inc, Chicago, IL, USA). The randomization process incorporates minimization and involves the consideration of three study variables for group stratification: sex (male/female), age (> 50/ ≤ 50 years), and crystal state (phakic eye/pseudophakic eye). A random allocation sequence will be created in advance and sealed in sequentially numbered opaque envelopes, allowing for randomization one at a time. Group allocation will be carried out by another researcher to ensure the randomization process is unbiased. All researchers involved in the study, including outcome assessors, statisticians, and data analysts, will be blinded to group assignment, but those providing the intervention will be informed as necessary. Before the trial, researchers will undergo comprehensive training in the randomization procedure and will be made aware of their responsibilities. The successful implementation and maintenance of the randomization and blinding methods will be validated to ensure the reliability and absence of bias in the trial results.

The investigator is strongly advised to uphold the blinding to the greatest extent possible. The actual allocation should not be revealed to the patient or any other study personnel, including site personnel, monitors, or project office staff. There should also be no written or verbal disclosure of the code in any of the patient-related documents associated with the study.

### Recruitment

At Dongyang People’s Hospital, more than 1000 patients seek treatment for vitreous floaters annually, ensuring an ample pool of participants for our studies. Participants will be recruited by ophthalmologists during outpatient visits, without any additional advertising. Interested patients will be invited to discuss the study details with an ophthalmologist. Those who meet all the inclusion criteria will receive complete information regarding their responsibilities and all procedures involved in the trial. Before enrollment, they will be asked to sign a written informed consent form. Insurance coverage, provided for all trial participants, is contracted to compensate for any harm that may occur during the final study visit.

### Intervention

#### YAG vitreolysis procedure

A Karickoff lens with goniosol will be used by the treating physician to perform YAG vitreolysis, with the number of shots determined based on clinical discretion. A focus offset may also be used if necessary, and the treatment will be conducted in single-shot mode with a maximum energy per pulse of 7 mJ. The endpoint of the treatment will be the vaporization of the Weiss ring into gas, as well as the fragmentation of any other vitreous opacities that are considered visually significant by the physician.

#### Sham laser procedure

The same procedure will be followed, but with the laser power turned down to 0.3 mJ and a separate lens covered by a filter that absorbs the power to ensure that no laser energy enters the eye.

### Primary outcome

The study will measure the outcomes at 3, 6, 9, and 12 months. These outcomes include the subjective improvement in floater symptoms, which is rated on a scale of 0 to 10. A score of 0 represents no symptoms, while a score of 10 indicates symptoms that significantly impact daily life. The study also measures the mean change in the National Eye Institute Visual Functioning Questionnaire-25 (NEI VFQ-25), which is a self-reported questionnaire that evaluates a patient’s visual function and its effect on their quality of life. The VFQ-25 consists of 25 items, which assess different domains of vision-related quality of life, including general vision, near and distance activities, driving, social functioning, and mental health. The minimum score for the VFQ-25 is 0, indicating the worst possible visual function and quality of life, while the maximum score is 100, representing the best possible visual function and quality of life. Higher scores on the VFQ-25 indicate better outcomes, indicating that the patient has improved visual function and quality of life. To address multiple comparisons in our study, we applied a Bonferroni correction. This correction method adapts the significance level to maintain an overall alpha level of 0.05, effectively controlling for the heightened risk of type I error associated with multiple testing.

### Secondary outcome

The secondary outcomes of the study encompass the objective assessment of changes through OCT and fundus photography. The objective evaluation of vitreous floater improvement using OCT and fundus photography is categorized into five levels: worsening, no change, mild improvement, significant improvement, and complete improvement. The objective evaluation will be conducted at 3, 6, 9, and 12 months.

Other secondary outcomes include the incidence and severity of ocular and systemic adverse events, such as retinal tears, retinal hemorrhage, retinal detachment, lens damage, and other related adverse events. The incidence rates of these adverse events will be recorded immediately after each laser treatment and at 1-month post-treatment.

### Sample size calculation

The sample size is determined a priori based on calculations assuming a modest improvement of 30% in symptoms in the YAG group compared to 10% in the sham group [7, 15]. This results in a sample of 66 patients with a standard deviation of 25%, an alpha level of 0.05, and a statistical power of 0.9. To account for a 5% rate of lost follow-up, it was decided to include 70 subjects in the study.

### Data collection, management, and monitoring

Before the trials commence, all investigators undergo comprehensive training on the clinical trial protocol, data management, and indicator evaluation methodology. Throughout the study, investigators diligently collect and record data in the participants’ medical records. All adverse events are carefully documented in the electronic case report form (CRF).

Participants who cannot be followed up for the entire study duration are considered dropouts. Participants have the option to withdraw from the study voluntarily in cases of intolerable side effects or poor treatment efficacy. Additionally, participants can withdraw from the study at any time for any reason. The investigators have the authority to withdraw participants from the study to prioritize their safety.

Any modifications to the study protocol require prior approval from the institutional ethics committee at Dongyang People’s Hospital. Once approved, these changes must be documented in the trial registry and subsequently included in the final research data report. Significant protocol amendments will be effectively communicated to trial participants through various channels, including written notifications, verbal explanations, or informational sessions. Participants will be given opportunities to seek clarification, ask questions, or express any concerns they may have.

During the experiment, concurrent treatments such as chronic diabetes, hypertension, anxiolytics, and antidepressant drugs are accepted. After all the follow-ups, participants are asked about receiving any other interventions during the study period, and this information is reported in the study results.

To minimize data loss, all participants are provided with guidance when they sign the informed consent form and commit to attending the scheduled treatment dates. Participants receive an appointment card to attend sessions. An evaluator is responsible for notifying and monitoring the participants every week (via telephone contact, WeChat, and/or email) and accompanying them during the research.

To ensure data quality control, the data manager conducts regular and timely data monitoring. When adding a new patient to the database, their identifying data is recorded on a printed form, which is not stored on the server. On this form, the participant’s name is represented by a combination of four English letters (the initials of their Chinese name pronunciation). This form is securely stored in a locked space, accessible only to the principal investigator, and may be utilized to reveal personal data if the need arises for unblinding purposes. Access to the final dataset is restricted solely to the principal investigator and the statistician, ensuring confidentiality and maintaining the integrity of the data. The data management team will maintain ongoing communication with the investigators regarding the progress of the trial, data consistency, instances of missing data, and any violations of time windows. If needed, queries for missing data and requests for clarification of inconsistencies or discrepancies will be issued.

The study will be overseen by the Data and Safety Monitoring Board (DSMB), comprising physicians, ethicists, medical statisticians, and a clinical manager. Their role involves regular monitoring, scheduled every 3 months. The DSMB will review safety data and clinical effectiveness reports to make informed decisions on whether the clinical trial should proceed.

### Statistics analysis

A comprehensive analysis is performed to delineate the demographic and clinical characteristics of the patient population. The normality of the variables is assessed using the Shapiro–Wilk test. The evaluated variables are depicted in tabular form, displaying both absolute and relative frequency distributions. Associations are examined utilizing either Pearson’s chi-square test or Fisher’s exact test, as warranted. The statistical significance of mean differences among quantitative variables is assessed through the paired and unpaired *t*-student tests. To assess variations across different time points within a group, the analysis of variance (ANOVA) with repeated measures is employed.

Continuous data are presented as mean ± standard deviation (SD), while categorical data are represented as counts (percentages). All analyses follow a two-tailed approach, with a significance level of 0.05. The statistical software package SPSS 17 (IBM Corporation, Armonk, NY) is employed for conducting data analysis, by established procedures.

In instances of data discontinuity, missing data will be handled by the “intention-to-treat” principle for conducting inferential statistical analysis. Missing data will be addressed through a dual strategy involving the last observation carried forward (LOCF) method and multiple imputation (MI). Additionally, sensitivity analyses will be conducted to evaluate the influence of various imputation methods on the study outcomes.

## Discussion

The objective of this study is to evaluate the efficacy and safety of YAG laser vitreolysis in the management of symptomatic vitreous floaters and to compare the disparities in both efficacy and safety between early and delayed YAG laser vitreolysis. Previous studies demonstrated that YAG laser vitreolysis was a potential treatment for vitreous floaters [8, 17, 18]. The findings from these randomized controlled trials have demonstrated the effectiveness of YAG laser vitreolysis in alleviating symptoms associated with vitreous floaters, with minimal occurrence of adverse effects. However, participants in these trials typically required the vitreous floater to remain stable for an extended period, often lasting 6 months or more. To the best of our knowledge, this study is the first randomized controlled double-blind trial that compared the safety and efficacy of early versus delayed YAG laser vitreolysis for vitreous floaters.

Vitreous floaters were usually recommended for observation [2]. Upon diagnosis, patients expressing concerns about floaters are commonly treated conservatively with reassurance and the expectation that, with time, they will adapt to the visual symptoms, or that the floaters will settle inferior to the visual axis. Nevertheless, some vitreous floaters can cause significant visual symptoms, such as a 67% decrease in contrast sensitivity in patients [19]. This results in a decline in health-related quality of life and significant anxiety among patients [5]. Therefore, some patients have a strong need to seek a solution early in the disease. In contemporary practice, the primary interventions for treating floaters are pharmacological vitrectomy [20] and Nd: YAG laser vitreolysis [21]. In the past, vitrectomy was generally considered to have higher efficacy than laser vitreolysis [22]; however, it posed potential risks to the retina, including retinal tears following the surgical procedure and the development of cataracts shortly after surgery [23, 24]. Given that laser vitreolysis is a less invasive procedure, it undoubtedly represents a better choice for patients with vitreous floaters who require early treatment. At present, there is a noticeable absence of relevant studies concerning early laser vitreolysis. Consequently, we conducted this trial to elucidate the efficacy and safety of early laser vitreolysis for vitreous floaters.

## Trial status

This is the first version of the protocol 2023.01 (published 19.09.2023). Recruitment for the study commenced in November 2022 and was initially planned to conclude by December 2023. However, due to the slow pace of recruitment, it was extended to conclude by December 2024. As of 19 September 2023, a total of 32 participants had been enrolled.

### Supplementary Information

**Additional file 1. **

## Data Availability

The study findings will be extensively shared through publications in open-access journals and presentations at conferences, both nationally and internationally. The data supporting the study results can be obtained from the corresponding author upon a reasonable request.

## Abbreviations

NEI VFQ-25: National Eye Institute Visual Functioning Questionnaire 25
OCT: Optical coherence tomography
CRF: Case report form
ANOVA: Analysis of variance
SD: Standard deviation
